# Indents

**Published:** 1904-05-15

**Authors:** 


### INDENTS.

☞ **Brief Articles of Technical or Scientific Value will receive notice and comment. Contributions invited.**

**Dental Graduates.** The Philadelphia Dental College has graduated 2,773 students, 350 of whom were from foreign countries. They are scattered throughout the world, Canada has 94, Germany 66, Austria 45, and so on down to Syria with only one.

**Polishing Vulcanite Plate.** Use aqua ammonia to mix the whiting instead of water, and the result will be quite satisfactory. After polishing wash the plate in tepid water and finish with a clean buff wheel.—W. J. Robinson, in *Stomatologist*.

**Forma-percha: Blair's.** I have used this preparation with considerable satisfaction as a root-canal filling and dressing for the past year. It is composed of pink base plate dissolved in eucalyptus, to which is added oil of cassia and paraform.—O. E. Inglis, in *Items of Interest*.

**Alcohol in Porcelain Work.** Moisten porcelain bodies in pure alcohol. The alcohol rapidly evaporates and does not generate steam and so cause porous porcelain. The porcelain so mixed will produce denser bakings and colors to correspond with those of the shade guides.—J. L. Helmer, in *Items*.

**Mouldine, To soften.** Break into small pieces and put in a suitable vessel and cover with enough water to make a solution. Add a teaspoonful of glycerine and let the vessel stand in a warm place until the water all evaporates, when it will be found that the glycerine has thoroughly permeated the mass of the clay and it is in good condition for manipulating. P. W. Smith, in *Stomatologist.*

**Orthoform Cotton.** Make a saturated solution of orthoform in alcohol and saturate cotton with this solution. Dry and put in a convenient bottle for use. It is very useful to pack the alveoli after extraction to prevent pain, also other wounds that are liable to be painful, such as pyorrhœa pockets, exposed pulps, lacerated gums after extracting impacted third molars, etc. It stops pain and hemorrhage and leaves no bad after-effects.

**Crowning a Dog's Tooth.** H. Watson showed before the Odonto-Chirurgical Society of Scotland a case of where a dog had fractured its canine tooth half way down the crown exposing the pulp, and causing the dog much discomfort. A cap crown was made, the pulp capped with formagen, and the crown cemented in place. The dog immediately resumed normal activities and no bad results have appeared after fifteen months.—*Record.*

**Wood-handled Dental Instruments Undesirable.** It is a mistake to have scalers and excavators or other dental instruments, which are liable to become infected from use in the mouth, made in part of wood, as they cannot be thoroughly sterilized except by steaming or soaking in chemical solution for such time as will soon destroy them. Where weight is an objection, aluminum handles should be substituted.—J. A. Woods, in *Dental Record.*

**Obtundent for Sensitive Dentin.** Apply adrenalin chloride, 1-1000, on a pellet of cotton for a few moments. Remove and place full strength formalin on another pellet of cotton, to which apply a suitably-sized *hot* ball burnisher. After about half a minute the most sensitive dentin can be comfortably excavated. Of course, care must be taken to protect the patient's eyes and air passages from the formaldehyde fumes.—Dr. W. Kay, in *Stomatologist.*

**Tooth Soap.**

| | |
|---|---|
| Castile soap, white | parts 225 |
| Chalk, precipitated | parts 225 |
| Orris root, powdered | parts 225 |
| Sugar, powdered | parts 112 |
| Rose water | parts 112 |
| Water, distilled | sufficient |
| Peppermint oil | parts 7 |
| Clove oil | parts 4 |

Dissolve the soap in water by aid of gentle heat, let cool down and add the rose water. Mix the chalk, orris root and sugar, and rub up the oils in the mixture. Add the soap mixture gradually, rubbing continuously. Put into suitable cups or jars.—*Jour. de Parfumerie.*

**Diagnosis of Pulp Sensitivity.** Heat a broad-faced amalgam instrument, take upon it a piece of gutta-percha high grade preferred on account of its ability to retain the heat, hold it in the flame until nearly ready to burn, then touch the tooth, gently at first, and you will immediately determine the relative sensitiveness of the pulps of the different teeth. If pulpitis is present, the pain will not immediately subside upon the removal of the hot gutta-percha.—Dr. Beach, in *Forum.*

**Radium.** Scientists with well-developed bumps of curiosity are still delving in the mysteries of that wonderful substance radium. One has recently discovered that the remarkable light shining from it is due to the pressure of the rare gas helium. Another has ventured so far as to taste a small fraction of a grain of the bromide. He saw happy visions for some time afterward and found the substance to be a powerful stimulant, affecting both the heart and kidneys for several hours.—*Pacific Drug Review.*

**Impression Taking.** Faulty models are often the result of using an upper tray, the rim of which is so high that it forces the lip upward, carrying with it the gum over the alveolus of anterior portion of the arch. This portion of the arch is also often distorted by stiff plaster or modeling compound, which flattens or protrudes it. Especially when the direction of the force used in adapting the material is toward the front of the mouth instead of at right angles to the horizontal part of the palate.

**Salve for Cracked Hands.** The Pharmaceutische Zeitung gives the following preparation for the healing of cracked and fissured hands so frequent in those who till the soil, and are engaged in similar labors:

| | |
|---|---|
| Menthol | 15 parts |
| Salol | 20 parts |
| Olive Oil | 20 parts |
| Lanolin | 500 parts |

Mix. Rub on the affected parts morning and evening. The itching and pain subside at once, and healing is speedily set up.—*National Druggist.*

**Coloring Cements to Match the Tooth.** I am at present using a number of highly-colored lead pencils. To get the required shade, the pencil is passed a few strokes over the ground glass slab, the necessary amount of liquid is then poured on the slab and thoroughly incorporated with the pigments. The powder is then added and thoroughly mixed. An absolutely white oxid of zinc powder is best to start with as a basis, tinting with blue, green, or red pencils, shading with the black lead-pencil.—M. Belcher, *Dental Office and Laboratory.*

**Abuse of Cocain and Morphin.** In face of the fact that the medicinal uses of cocain, morphin and opium are said to have decreased, governmental statistics show that importations of these drugs have increased about 500 percent in the last eight years. The action of the American Pharmaceutical Association in drafting a model law to control the sale of such drugs was certainly timely, and it is sincerely to be hoped that the various States will not be slow in taking the matter up with their law-making bodies. —*Pacific Drug Review.*

**Obtunding Dentin with Sodium Dioxid.** Place the dry sodium dioxid in the cavity and add a little water, let stand for a brief time, during which the tooth will be likely to ache. Then dry thoroughly and it will be found that the dentin is insensible. For deep work a second application may be necessary. The dam should always be applied and the cavity well washed out before the filling is inserted. This remedy seems to act efficiently in badly irritable cases.—E. M. Soule, in *Items*.

**Muscular Paralysis from Extraction of a Tooth.** Mr. F. Rose related to the Liverpool Odontological Society that he had administered gas to a man 45 years old, who was taken with a violent spell during the anesthesia. When he recovered he complained of paralysis of his left arm. A surgeon found that some of the tendons of the shoulder muscles had been injured probably by great pressure of the elbow on the chair arm. It required a long time of a bandaged shoulder and rest to recover the use of the arm.—*Record*.

**Amalgam Inlay.** Burnish to the cavity a gold matrix in the usual manner. While the matrix is still in the tooth fill it with amalgam, and then remove it with its amalgam filling and set aside until the amalgam becomes hard. Invest it in plaster of paris so that its surface can be polished with disks, etc. Then insert it with cement as an ordinary porcelain inlay. Advantages: perfect adaptation to the cavity margins, an edge that will not disintegrate, no shrinkage, and a well-polished surface with restoration of contour.—Dr. Ottolengui in *International*.

**Fire in Pennsylvania Dental College.** February 23, 1904, a fire broke out under the amphitheatre and before it could be checked that portion of the building was badly damaged as well as the lecture room illustration material, consisting of charts, models, museum specimens, etc. As the fire occurred about three o'clock the egress from the clinical rooms was cut off and students and patients had to leave the building by the fire escapes. No one was injured. The building was fully insured. The work of the college was not long interrupted.—*Brief.*

**Method of Mixing Amalgam.** Arrange a piece of rubber dam over the palm of one hand and upon this place the alloy and mercury. Gather up the corners and edges of the rubber and twist together in the manner that a chamois skin would be used for the same purpose. Hold the twisted portion of the rubber with one hand, place the bulbous portion containing the alloy and mercury against a flat, smooth surface, and roll vigorously with the other hand. The advantages of this method will suggest themselves when tried.—Dr. R. L. Graber, *Review.*

**Why the Rubber Dam?** Very many times it is convenient and painful to apply the dam preparatory to the removal of the pulp under pressure anesthesia. The editor has long discarded the dam in cases where its employment will be troublesome or painful. This occurs very often, as in cases of pulp-exposure at the cervix of the tooth, the cavity well covered by the gum. Careful attention to absorption of the excess of cocain, and directions to eject all saliva, proves that the operation without the dam is perfectly safe, effective and advisable.—*Western Journal.*

**Veneering Vulcanite Plates.** Pack in the usual manner, open flask and if there should be all the rubber needed, take a sheet of the vulcanite used for veneer and stretch it until quite thin,and place it over the vulcanite in the flask, close flask and vulcanize. This can be used to produce beneficial and beautiful vulcanite plates. To line a plate for instance with black rubber, it can be done by stretching a thin sheet of black rubber over the surface of the case, or by painting the plaster model with a solution of black rubber, then pack the flask and vulcanize.—W. J. Robinson, in *Stomatologist.*

**Antikamnia and Codein for Neuralgia.** There are many painful conditions associated with the practice of dentistry which are not amenable to local applications, such as facial neuralgia, headache, earache and even toothache from hidden causes. They are also just as annoying to the patient as toothache from exposed pulps or irritated dental structures and demand treatment for relief. Since local medication is out of the question, resort must be had to systemic anodynes of the narcotic class to get permanent relief. Of the several preparations on the market, antikamnia is one of the most effective and easily prescribed. It has the advantage of leaving no secondary bad results when properly administered. In severe neuralgia the five-grain antikamnia and codein tablet administered one every hour until not more than three or four have been taken, will be found very helpful. They are also said to allay nervousness if given a short time before painful operations on the teeth.—*Exchange.*

